# *Sensor-Motor Maps* for Describing Linear Reflex Composition in Hopping

**DOI:** 10.3389/fncom.2017.00108

**Published:** 2017-11-27

**Authors:** Christian Schumacher, André Seyfarth

**Affiliations:** Lauflabor Locomotion Laboratory, Centre for Cognitive Science, Institute of Sport Science, Technische Universität Darmstadt, Darmstadt, Germany

**Keywords:** feedback pathways, hopping, motor control, functional decomposition, neuromechanics, multisensory integration, muscle-tendon function, *sensor-motor map*

## Abstract

In human and animal motor control several sensory organs contribute to a network of sensory pathways modulating the motion depending on the task and the phase of execution to generate daily motor tasks such as locomotion. To better understand the individual and joint contribution of reflex pathways in locomotor tasks, we developed a neuromuscular model that describes hopping movements. In this model, we consider the influence of proprioceptive length (LFB), velocity (VFB) and force feedback (FFB) pathways of a leg extensor muscle on hopping stability, performance and efficiency (metabolic effort). Therefore, we explore the space describing the blending of the monosynaptic reflex pathway gains. We call this reflex parameter space a *sensor-motor map*. The *sensor-motor maps* are used to visualize the functional contribution of sensory pathways in multisensory integration. We further evaluate the robustness of these *sensor-motor maps* to changes in tendon elasticity, body mass, segment length and ground compliance. The model predicted that different reflex pathway compositions selectively optimize specific hopping characteristics (e.g., performance and efficiency). Both FFB and LFB were pathways that enable hopping. FFB resulted in the largest hopping heights, LFB enhanced hopping efficiency and VFB had the ability to disable hopping. For the tested case, the topology of the *sensor-motor maps* as well as the location of functionally optimal compositions were invariant to changes in system designs (tendon elasticity, body mass, segment length) or environmental parameters (ground compliance). Our results indicate that different feedback pathway compositions may serve different functional roles. The topology of the *sensor-motor map* was predicted to be robust against changes in the mechanical system design indicating that the reflex system can use different morphological designs, which does not apply for most robotic systems (for which the control often follows a specific design). Consequently, variations in body mechanics are permitted with consistent compositions of sensory feedback pathways. Given the variability in human body morphology, such variations are highly relevant for human motor control.

## 1. Introduction

The redundancy of the musculoskeletal and neural systems poses a major challenge in human locomotion research. For instance, the motor control system may utilize different strategies for performing specific motions with redundancy in the body's physiology (e.g., many involved muscles), kinematics (e.g., redundant motion trajectories) (Bernstein, 1967), and neuromuscular control (e.g., recruitment of motor units) (Henneman et al., 1965) including neural networks in the spinal cord that contribute substantially to controlling rhythmic and repetitive motions. To date, it is unknown how the neuromuscular system explores and exploits the redundancy and how the different levels are organized and interconnected to achieve functionally relevant activation patterns (Donelan and Pearson, 2004b). Proprioceptive feedback and central pattern generators (CPGs) presumably generates appropriate motor control commands depending on the tasks and the phase of the motion (Dietz, 1992; Taube et al., 2012).

Similarly, computational approaches aiming at mimicking human activation patterns and motion trajectories must address the “redundancy problem” in motor control. Most commonly, these approaches reduce the degrees of freedom by specific neuromuscular structures or hierarchies (e.g., specific combinations of CPGs, sensory pathways etc.) that follow certain control policies or rules. For instance, Song and Geyer (2015) used multiple “spinal modules” (decentralized feedback control) coordinated by a supra-spinal layer to predict several gaits and generate robust behavior even after perturbations (Song and Geyer, 2017). Other studies used combinations of CPGs and proprioceptive feedback (modifying the central patterns) to generate appropriate activation patterns (Taga, 1998; Ogihara and Yamazaki, 2001; Hase et al., 2003; Paul et al., 2005). Moreover, muscle synergies (groups of synchronized co-contracting muscles during a motion) are used to reduce the dimensionality and thus the redundancy of the neuromuscular system (D'Avella et al., 2003; Bizzi et al., 2008). For instance, Ting et al. (2012) used a neuronal network for generating a muscle synergy driven balancing task based on center of mass (COM) kinematics.

In contrast to previous studies with a detailed representation of the neural networks (including their hierarchies), this study focused on integrating multiple sensory pathways at the elementary sensor-motor-level (Loeb, 1995) to determine how individual reflex pathways of muscle force (FFB), fiber length (LFB) and velocity (VFB) can support—in isolation and in combination—the repulsive leg function (Sharbafi and Seyfarth, 2017) during the stance phase of hopping (Haeufle et al., 2012). By blending individual sensory pathways, we investigated the capacity of the neuromuscular feedback system to generate goal-directed motions. We visualized and evaluated the space in which the monosynaptic reflex system can operate to generate functional motions (for generating stable, performant or efficient hopping). We call these reflex parameter spaces *sensor-motor maps* and suggest that studying their topology can be used to explore the redundancy of multisensory integration. This approach differs from previous approaches because the general concept of such *sensor-motor maps* only relies on a few primitive assumptions on the neuromuscular structure. The topologies of these *sensor-motor maps* reflect the task-specific contributions of the sensory pathways that are moderated by the mechanical interaction of the locomotor system with the environment. Our overall goal was to identify enabling and disabling pathways for individual locomotor functions. We expected that several different pathways may generate stable hopping, but that pathway-specific features determine hopping performance and efficiency.

To show the general validity of our approach we varied parameters of the environment (ground compliance) and the body morphology: compliance (tendon elasticity), geometry (segment lengths) and inertia (body mass). Furthermore, we explored the sensitivity of the model to variations of feedback and model parameters.

## 2. Materials and methods

To focus on the integration of different sensory pathways, we considered a highly simplified muscle-driven model allowing the evaluation of motion execution with respect to stability, performance and efficiency. Therefore, we used the hopping model by Geyer et al. (2003) with idealized sensory receptors and motorneurons capturing the basic neural control principles with the least possible system complexity (Full and Koditschek, 1999; Brown and Loeb, 2000; Pearson et al., 2006). We chose the signals of three muscle receptors (muscle force of Golgi tendon organs, fibre length and fibre velocity of muscle spindles) to focus on local proprioceptive circuits.

### 2.1. Mechanical hopping model

**Figure 1:**
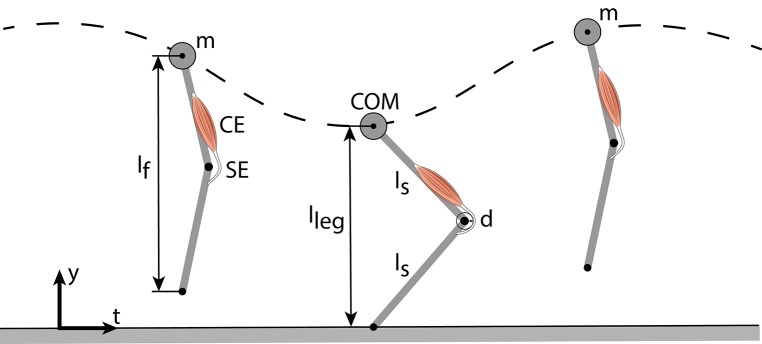
Vertical hopping model (Geyer et al., 2003) comprising a point mass (*m*), two massless leg segments and a leg extensor muscle-tendon-complex (MTC), consisting of a contractile element (CE) and a serial elastic element (SE). During flight phase, the leg flight length (*l*_*f*_) stays constant. In stance, the MTC generates a pulling force that acts on the lever arm (*d*) which creates an extension torque.

The model of Geyer et al. (2003) consists of a point mass *m* (center of mass, COM) and two massless segments (length *l*_*S*_) representing the thigh and shank (Figure 1). The leg length during flight (*l*_*f*_) is held constant until the vertical COM height equals the flight leg length (touch-down). During stance, a muscle-tendon-complex (MTC) modeling the knee extensors counteracts the gravitational force (gravitational constant *g*). The MTC consists of a contractile element (CE) and a serial elastic element (SE) (Equations 1, 2). Take-off occurs when the leg force vanishes or when the vertical displacement of the point mass exceeds the flight leg length.

(1) $$\begin{array}{r} l_{MTC}=l_{CE}+l_{SE} \end{array}$$

(2) $$\begin{array}{r} F_{MTC}=F_{CE}=F_{SE} \end{array}$$

The length of the MTC is defined by a reference length (*l*_*MTC,ref*_), a corresponding reference knee angle (φ_*ref*_) and the knee lever arm (*d*): *l*_*MTC*_ = *l*_*MTC,ref*_ − *d* (φ − φ_*ref*_). The force of the CE is calculated as *F*_*CE*_ = *F*_max_^*^ *f*_l_^*^ *f*_v_^*^ *ACT* using the maximum isometric force (*F*_*max*_), force-length-relationship (*f*_*l*_), force-velocity-relationship (*f*_*v*_) and activation state of the contractile element (*ACT*, see Equation 11). The force-length-relationship and force-velocity-relationship are implemented by non-linear approximations (Geyer et al., 2003):

(3) $$\begin{array}{r} f_{l}\left(l_{CE}\right)=exp\left(c|\frac{l_{CE}-l_{opt}}{l_{opt}w}{|}^{3}\right) \end{array}$$

(4) $$\begin{array}{r} f_{v}\left(v_{CE}\right)=\left\{ \begin{array}{cc} N+\left(N-1\right)\;\frac{v_{max}\;-\;v_{CE}}{7.56\;K\;v_{CE}\;-\;v_{max}}\; & v_{CE}\geq 0\\ \frac{v_{max}\;-\;v_{CE}}{v_{max}\;+\;K\;v_{CE}}\; & v_{CE}<0 \end{array}\right. \end{array}$$

These equations use a width (*w*) and a curvature constant (*c*) of the force-length-curve as well as optimum length of the CE (*l*_*opt*_), eccentric force enhancement (*N*), maximum shortening velocity (*v*_*max*_) and a second curvature constant (*K*). The force-length-relationship values can range from 0 to 1. The force-velocity-relationship values can range from 0 to 1 for concentric contractions and from 1 to 1.5 for eccentric contractions (because of the eccentric force enhancement *N*). To define the serial elastic element in the MTC a progressive force-length dependency (Equation 5) was used (van Ingen Schenau, 1984). Therefore, the reference strain (ε_*ref*_) determines the relation of the force acting on the serial element and its corresponding stretch in relation to its rest length (*l*_*rest*_) (Equation 6).

(5) $$\begin{array}{r} f_{SE}\left(\varepsilon \right)=\left\{ \begin{array}{cc} {\left(\varepsilon /\varepsilon_{ref}\right)}^{2}\; & \varepsilon >0\\ 0\; & \varepsilon \leq 0 \end{array}\right. \end{array}$$

(6) $$\begin{array}{r} \varepsilon =\left(\frac{l_{SE}}{l_{rest}}\right)-1 \end{array}$$

### 2.2. Extension of the neuromuscular model

**Figure 2:**
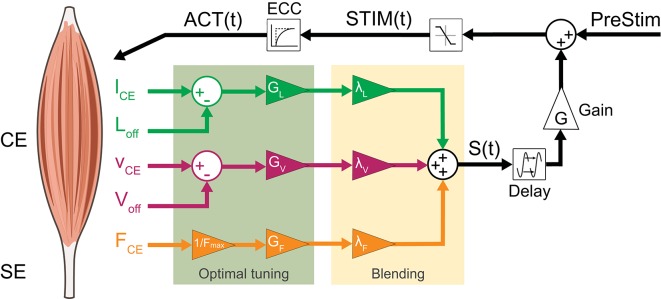
Neuromuscular reflex model which fuses Ia afferent signals (*l*_*CE*_, *v*_*CE*_ and offsets *L*_*off*_, *V*_*off*_) and normalized Ib afferent (*F*_*CE*_) pathways: All three sensory signals are gained (*G*_*L*_, *G*_*V*_, *G*_*F*_ ≥ 0) and weighted (λ_*i*_). The resulting summation signal *S*(*t*) is then delayed (Δ_*S*_) and gained (*G* ≥ 0). This signal is then added to a constant pre-stimulation value to mimic a positive excitatoric postsynaptic potential at the α-motoneuron. The stimulation signal *STIM(t)* is confined to values between 0 and 1 and delayed by the excitation-contraction-coupling (ECC) resulting in the activation signal *ACT(t)* of the CE.

To consider fused feedback pathways, we extended the neuromuscular feedback model by a linear combination of muscle force (FFB), fibre length (LFB) and fibre velocity feedback (VFB) pathways (Figure 2). All three afferent pathway signals are multiplied by a blending factor λ_*i*_ weighting the individual pathways resulting in the summation signal *S*(*t*) (Equation 7) where *G*_*i*_, *F*_*max*_, *L*_*off*_ and *V*_*off*_ denote the individual gains, maximum isometric force and offsets of length and velocity pathways, respectively. By restricting the sum of all blending factors (Equation 8), one weight can always be calculated from the other two (Seyfarth et al., 2001).

(7) $$\begin{array}{r} \begin{array}{c} S\left(t\right)=\lambda_{F}*G_{F}*F_{CE}/F_{max}\\ +\lambda_{L}*G_{L}*\left(l_{CE}-L_{off}\right)\\ +\lambda_{V}*G_{V}*\left(v_{CE}-V_{off}\right) \end{array} \end{array}$$

(8) $$\begin{array}{r} \lambda_{F}+\lambda_{L}+\lambda_{V}=1,\;0\leq \lambda_{F,L,V}\leq 1 \end{array}$$

**Figure 3:**
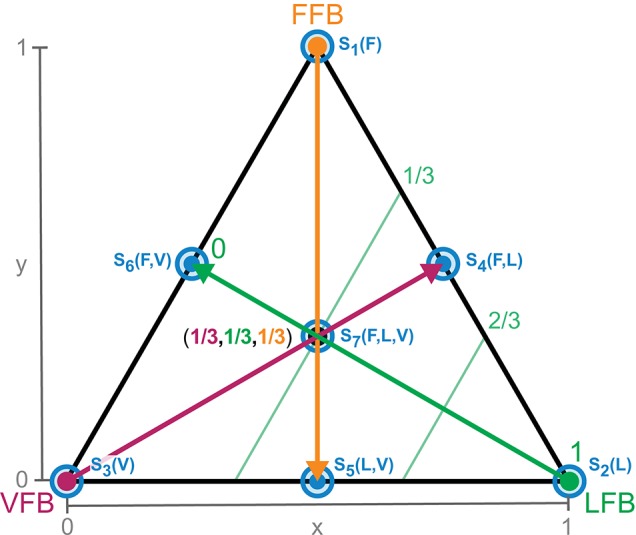
Schematic explanation of sensory pathway blending through parameter reduction (Equation 8). Corners of the triangle represent a full contribution of one single feedback (e.g., 0% VFB, 0% FFB, 100% LFB). Every point within the triangle (coordinates *x* and *y*) represents a unique combination of the three feedback pathways. Arrows (thick) and contour lines (thin) explain the blending of feedback pathways. The larger the distance of a point to a corner (e.g., LFB) the smaller the contribution of that feedback pathway in the blended signal. Exemplary, the middle point describes an equal composition of all feedback pathways (1/3 VFB, 1/3 FFB, 1/3 LFB). Optimal tuning of the individual feedback pathways is done by optimization for full contribution of only one sensory reflex (corners). These optimal reflex pathways are used for all compositions (see section 2.3). The blue circles (*S*_1_(*F*) to *S*_7_(*F,L,V*)) denote the specific compositions of feedback pathways used for the sensitivity analysis (see section 2.3.8).

This normalizes the blending of individual contributions and reduces the dimensionality by projection onto a two-dimensional space (of independent blending factors). Triangles visualize all possible (projected) feedback compositions (Figure 3). The corners of the triangle represent the isolated individual feedback pathways (purely FFB, LFB, or VFB). Every point within the triangle represents a blending of the individual feedback pathways and refers to two-dimensional Cartesian coordinates (space *V*, *x*, and *y* between 0 and 1). The projection to the blending factors (space *W*) is described by: *f* : *V* → *W* (Equation 9). Hence, the larger the distance of a point to a corner (e.g., VFB) the smaller is the contribution of that specific feedback pathway in the blended signal *S*(*t*). Individual feedback pathways in isolation (corners) are parameterized by optimization (see section 2.3).

(9) $$\begin{array}{r} f=\left\{ \begin{array}{cc} x\to \lambda_{L}\; & 0\leq x\leq 1\\ y\to \lambda_{F}\; & 0\leq y\leq 1 \end{array}\right. \end{array}$$

After blending, the proprioceptive signal (*S*(*t*)) is delayed by Δ_*S*_, gained by *G* and added to the stimulation bias (*PreStim*) (Equation 10). Then, this signal is confined to values between 0 and 1 and input into the excitation-contraction-coupling (Equation 11) described by a first-order differential equation resulting in the activation signal *ACT*(*t*) (Geyer et al., 2003; Haeufle et al., 2012).

(10) $$\begin{array}{r} STIM\left(t\right)=\left\{ \begin{array}{cc} PreStim\; & t<\Delta_{S}\\ PreStim+G*S\left(t-\Delta_{S}\right)\; & t\geq \Delta_{S} \end{array}\right. \end{array}$$

(11) $$\begin{array}{r} \tau \frac{dACT\left(t\right)}{dt}=STIM\left(t\right)-ACT\left(t\right) \end{array}$$

### 2.3. Model parameter and optimization

#### 2.3.1. Model parameters

Parameters of the mechanical model (Table 1) were taken from Geyer et al. (2003). The initial position of the point mass was set to 1.05 m and its initial velocity to 0 m/s.

**Table 1:** Parameters of the hopping model taken from Geyer et al. (2003).

**Parameter**	**Value**	**Unit**
Body mass *m*	80	[kg]
Gravitational constant *g*	9.81	[m/s^2^]
Initial body mass height *y*_0_	1.05	[m]
Flight leg length *l*_*f*_	0.99	[m]
Segment length *l*_*S*_	0.5	[m]
Knee lever arm *d*	0.04	[m]
MTC reference length *l*_*MTC,ref*_	0.5	[m]
Reference knee angle φ_*ref*_	110	[°]
Maximum isometric force *F*_*max*_	22,000	[N]
Optimum length of CE *l*_*opt*_	0.1	[m]
Curvature constant of *f*_*l*_ *c*	0.05	[ ]
Width *w*	0.4	[ ]
Maximum shortening velocity *v*_*max*_	–12	[m/s]
Eccentric force enhancement *N*	1.5	[ ]
Curvature constant of *f*_*v*_ *K*	5	[ ]
SE rest length *l*_*SE, rest*_	0.4	[m]
“Stiff” reference strain of SE ε_*stiff*_	0.01	[ ]
“Moderate” reference strain of SE ε_*moderate*_	0.03	[ ]
“Compliant” reference strain of SE ε_*compliant*_	0.05	[ ]
“Heavy” body mass *m*_*heavy*_	96	[kg]
“Moderate” body mass *m*_*moderate*_	80	[kg]
“Light” body mass *m*_*light*_	64	[kg]
“Long” segment length *l*_*S,long*_	0.6	[m]
“Moderate” segment length *l*_*S,moderate*_	0.5	[m]
“Short” segment length *l*_*S,short*_	0.4	[m]
“Stiff” ground stiffness *k*_*stiff*_	9,999	[kN/m]
“Moderate” ground stiffness *k*_*moderate*_	500	[kN/m]
“Compliant” ground stiffness *k*_*compliant*_	100	[kN/m]
Excitation-contraction time constant τ	0.01	[s]
Feedback signal time delay Δ_*S*_	0.015	[s]

#### 2.3.2. Optimization of feedback parameters

To identify feedback parameters of the extended neuromuscular model (optimal tuning part in Figure 2), a pattern search optimization algorithm was used (Torczon, 1997). The pattern search algorithm was implemented to search for parameters sets that result in stable hopping patterns (more than *n* = 50 steps, first criterion). As second optimization criterion, the maximum height of the body mass *h*_*max*_ = *y*_*max,apex*_ for steady-state hopping (*n* = 49 step) was chosen. Simulations were checked for steady-state motion. Optimizations of all individual feedback pathways (in isolation) were done for “stiff tendon” and “rigid ground” and repeated five times each with random starting points to avoid finding local maxima. The limits of parameter values were 0.1 ≤ *G*_*F*_ ≤ 3 (FFB), 0.1 ≤ *G*_*L*_ ≤ 200 and 0 ≤ *L*_*off*_ ≤ 3 (LFB) as well as 0.1 ≤ *G*_*V*_ ≤ 3 and −1 ≤ *V*_*off*_ ≤ 0 (VFB) aligned to results from Geyer et al. (2003). The best performing solution was used for further simulation and analysis.

#### 2.3.3. Simulation and optimization environment

Simulations and optimizations were implemented in Matlab Simulink (release 2016b, Mathworks, Natick, Massachusetts, USA). For the simulations, the variable-step solver “ode45” with relative and absolute tolerances of 10^−8^ was used. Optimization was done using the Global Optimization Toolbox (Version 3.4.1).

#### 2.3.4. Tendon elasticity changes

To change the SE elasticity, three configurations for the reference strain were used: (1) “stiff tendon” (ε_*stiff*_ = 0.01), (2) “moderate tendon” (ε_*moderate*_ = 0.03), and (3) “compliant tendon” (ε_*compliant*_ = 0.05). For equal forces, smaller reference strain values indicated less associated stretch and thus a stiffer length-force dependency of the SE. These SE elasticity levels are in a range used by other simulation studies (Pandy et al., 1990; Bobbert, 2001; Nagano et al., 2004).

#### 2.3.5. Body mass changes

The body mass of the model (*m*) was varied to 80 and 120% of the original body mass (80 kg): (1) “light mass” (*m*_*light*_ = 64 kg), (2) “moderate mass” (*m*_*moderate*_ = 80 kg), and (3) “heavy mass” (*m*_*compliant*_ = 96 kg).

#### 2.3.6. Segment length changes

The leg geometry was altered by changing the length of both segments (*l*_*S*_) to 80 and 120% of the original segment length (0.5 m): (1) “short segments” (*l*_*S,short*_ = 0.4 m), (2) “moderate segments” (*l*_*S,moderate*_ = 0.5 m), and (3) “long segments” (*l*_*S,long*_ = 0.6 m). To keep the take-off conditions and energy level of the system consistent for all segment length configurations, the initial body mass height (*y*_0_ = 2 ∗ *l*_*S*_ + 0.05 m) and the flight leg length (*l*_*f*_ = 2 ∗ *l*_*S*_ − 0.01 m) were adjusted accordingly.

#### 2.3.7. Ground compliance changes

To modulate the vertical ground stiffness, the model was slightly adapted. During stance, a linear spring constant (*k*_*ground*_) and the leg force or vertical ground reaction force (*F*_*leg*_) define the foot position (*y*_*FP*_):

(12) $$\begin{array}{r} y_{FP}\left(F_{leg}\right)=\left\{ \begin{array}{cc} y_{COM}-l_{f}\; & during\;flight\\ -\frac{F_{leg}}{k_{ground}}\; & during\;stance \end{array}\right. \end{array}$$

The foot position during stance can only reach values ≤ 0 because the take-off condition is met for vanishing leg force (*F*_*leg*_ < 0). To change the ground compliance, three configurations for the spring constant were chosen: (1) “compliant ground” (*k*_*compliant*_ = 100 kN/m), (2) “moderate ground” (*k*_*moderate*_ = 500 kN/m), and (3) “stiff ground” (*k*_*stiff*_ = 9, 999 kN/m). These ground stiffness values are in a range used by other computational or experimental studies (Farley et al. (1998): 20–35,000 kN/m, Moritz and Farley (2004): 27–411 kN/m, van der Krogt et al. (2009): 75–3,100 kN/m).

#### 2.3.8. Sensitivity analysis

To evaluate the performance of the model for different parameter settings, we analyzed its parametric sensitivity. We randomly altered feedback parameters (*G*_*F*_, *G*_*L*_, *L*_*off*_, *G*_*V*_, *V*_*off*_, *PreStim*, Δ_*S*_) as well as model parameters (ε_*ref*_, *l*_*S*_, *m*). Physiological parameters were normally distributed (*l*_*S*_: μ = 0.5 m, σ^2^ = 0.02 m; *m*: μ = 80 kg, σ^2^ = 5 kg) whereas other parameters were uniformly distributed (1 ≤ *G*_*F*_ ≤ 5; 100 ≤ *G*_*L*_ ≤ 160; 0.06 m ≤ *L*_*off*_ ≤ 0.1 m; 1 ≤ *G*_*V*_ ≤ 5; −1*m*/*s* ≤ *V*_*off*_ ≤ 0 m/s; 0.01 ≤ *PreStim* ≤ 0.2; 0.01 s ≤ Δ_*S*_ ≤ 0.05 s; 0.01 ≤ ε_*ref*_ ≤ 0.05). For the sensitivity analysis, the ground stiffness remained unchanged (no compliance). Because parametric influences differ depending on the feedback blending, we considered seven reflex pathway compositions for our sensitivity analysis. Figure 3 shows the location of these seven compositions (*S*_1_(*F*) to *S*_7_(*F,L,V*)). For each composition, *n* = 1,000 simulations with randomized parameters were performed. Maximum hopping height (Δ*h*_*max*_) and hopping efficiency (η) were calculated as performance measures (see section 2.4.2). The sensitivity of these variables was further tested with SPSS 24.0. (IBM Corporation, Armonk, New York, USA). Spearman's rho correlation coefficients (*r*) with significance values (two-sided test) and standardized regression coefficients (β) were calculated for simulations that resulted in stable hopping. Correlations were considered to be moderate for 0.5 ≤ *r* < 0.7 (−0.5 ≥ *r* > −0.7) or high for *r* ≥ 0.7 (*r* ≤ −0.7) if *p*-values were significant (*p* < 0.01).

### 2.4. Performance metrics

Depending on the force generated during stance, the predicted motion will result in continuous and stable hopping or in a bound motion (leg remains in contact to the ground) where the model lands but does not lift off. In case of hopping, the blending compositions were evaluated by calculating the following metrics.

#### 2.4.1. Stability criterion

To determine if the extended neuromuscular reflex model will result in stable hopping or bound motion, we examined the number of steps to fall, and simulations resulting in at least 50 steps were considered stable.

#### 2.4.2. Hopping metrics

The following criteria were used to evaluate the performance of the hopping model with respect to energetics and motion dynamics for the last step (n = 49) of the simulation. Simulations were checked for steady-state motion.

1. The model generates a motion performance or a mechanical output during hopping defined as the steady-state vertical hopping height of the body mass (Δ*h*_*max*_ = *y*_*apex*_ − *l*_*f*_) at the instance of apex (*v*_*y,apex*_ = 0). During flight, the system energy is equivalent to the potential energy at the apex: *E*_*system*_ = *m g h*_*max*_.
2. To describe the hopping motion we calculated the hopping frequency (*f*_*hop*_) and the effective stiffness of the leg *k*_*leg*_ = *F*_*leg,max*_/Δ*l*_*leg,max*_.
3. Because the tendon and muscle share the same force (Equation 2), knowledge about the relative work generation (and length deflection) of the CE and the MTC is of interest. Hence, we calculated the maximum amount of work generated by the CE (*W*_*CE,max*_) relative to its equivalent of the whole MTC (*W*_*MTC,max*_) that was then simplified to the ratio of the maximum deflection of both elements with respect to the elements' rest lengths:
  (13) $$\begin{array}{r} \alpha =\frac{W_{CE,max}}{W_{MTC,max}}=\frac{F_{MTC,max}*\Delta l_{CE,max}}{F_{MTC,max}*\Delta l_{MTC,max}}=\frac{\Delta l_{CE,max}}{\Delta l_{MTC,max}} \end{array}$$
  This factor describes the maximum amount of work produced in the muscular element relative to the overall maximum contribution of the MTC.
4. To evaluate the metabolic effort of the CE, we used the velocity-dependent metabolic cost model by Minetti and Alexander (1997) and Robertson and Sawicki (2014) favoring eccentric contractions with reduced metabolic effort (Equation 15). This is scaled by the activation signal during ground contact (*ACT*(*t*)), maximum isometric Force (*F*_*max*_) and maximum shortening velocity (*v*_*max*_) to calculate the metabolic rate (*M*_*eff*_(*t*)) (Krishnaswamy et al., 2011; Robertson and Sawicki, 2014):
  (14) $$\begin{array}{r} M_{eff}\left(t\right)=\Phi \left(v_{CE}\right)\;*\;ACT\left(t\right)\;*\;|F_{max}*v_{max}| \end{array}$$
  (15) $$\begin{array}{r} \Phi \left(v_{CE}\right)=\left\{ \begin{array}{cc} 0.23-0.16*e^{\left(-8*\frac{v_{CE}}{v_{max}}\right)}\; & v_{CE}\geq 0\\ 0.01-0.11*\frac{v_{CE}}{v_{max}}+0.06*e^{\left(8\;*\;\frac{v_{CE}}{v_{max}}\right)}\; & v_{CE}<0\\ \; \end{array}\right. \end{array}$$
  We derived the averaged metabolic effort (${\bar{M}}_{eff}$) per hopping cycle by an integration of the metabolic rate (*M*_*eff*_(*t*)) during ground contact and a normalisation with the body mass (*m*) and the contact time (*T*_*contact*_) (Robertson and Sawicki, 2014):
  (16) $$\begin{array}{r} {\bar{M}}_{eff}=\int_{0}^{T_{contact}}M_{eff}\left(t\right)dt/\left(m*T_{contact}\right) \end{array}$$
5. Hopping efficiency was quantified as the ratio of hopping height (Δ*h*_*max*_) (output) to averaged metabolic effort of the CE (input): $\eta =\frac{\Delta h_{max}}{{\bar{M}}_{eff}}$.

## 3. Results

### 3.1. Individual hopping patterns

**Figure 4:**
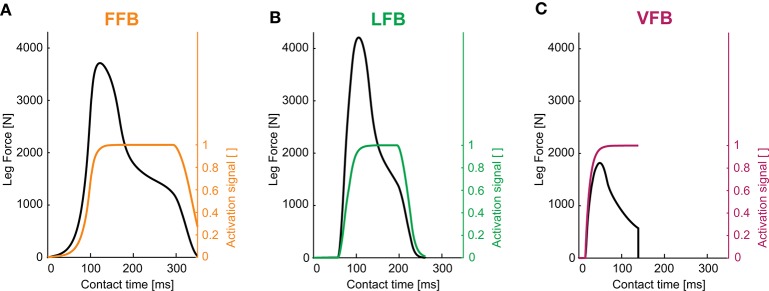
Leg forces and activation signals during one stance phase for optimized feedback parameters of individual pathways (*y*_0_ = 1.05 m, *G* = 1, *PreStim* = 0.01, ε_*stiff*_ = 0.01, rigid ground): **(A)** force feedback (*G*_*F*_ = 2.6), **(B)** length feedback (*G*_*L*_ = 130, *L*_*off*_ = 0.08) and **(C)** velocity feedback (*G*_*V*_ = 2.9, *V*_*off*_ = −0.6). Maximum hopping heights are Δ*h*_*max*_ = 0.126 m (FFB), Δ*h*_*max*_ = 0.063 m (LFB) and Δ*h*_*max*_ = 0.002 m (VFB).

The optimization of the individual feedback pathways (with ε_*stiff*_ = 0.01) resulted in neuromuscular model parameters that produced a maximum hopping height for stable hopping patterns (Table 2). For these feedback parameters, FFB was the best performing optimization with a hopping height of 0.126 m. The maximum hopping height of LFB was 0.063 m. VFB did not produce a high performance with a maximum hopping height of 0.002 m just above the flight leg length. The predicted leg forces and activation signals are shown in Figure 4. The activation profile and subsequently the leg force profiles of FFB showed an increasing amplification. Compared to leg forces of FFB, LFB produced higher peak leg forces but shorter contact times. The rise of the LFB activation signal was delayed to the instance of touch-down by about 50 ms. Here, the length offset *L*_*off*_ suppressed the early activation signal (also reported by Geyer et al., 2003). VFB produced half the leg force and half the contact time compared to FFB and LFB reflected in the small hopping height (0.002 m). While FFB and LFB showed delayed increase in the leg force (more than 50 ms after touch-down), the VFB caused an almost instantaneous response in the activation signal resulting in high (eccentric) force generation and thus high energy losses during leg compression. The CE remained less stretched and started to shorten before reaching optimal fibre length (*f*_*l*_ < 0.2) limiting positive (concentric) muscle work during leg extension.

**Table 2:** Optimization results of individual feedback parameters (*y*_0_ = 1.05 m, *G* = 1, *PreStim* = 0.01, ε_*stiff*_ = 0.01, rigid ground).

**Parameter**	**Force feedback (FFB)**	**Length feedback (LFB)**	**Velocity feedback (VFB)**
Individual gain	*G*_*F*_ = 2.6	*G*_*L*_ = 130	*G*_*V*_ = 2.9
Individual offset	−	*L*_*off*_ = 0.08	*V*_*off*_ = −0.6
Maximum hopping height Δ*h*_*max*_	0.126 m	0.063 m	0.002 m

### 3.2. *Sensor-motor maps*

The following section describes the results of the blended feedback pathways and the *Sensor-motor maps* for different motion characteristics (e.g., hopping stability, performance and efficiency).

#### 3.2.1. Hopping stability

**Figure 5:**
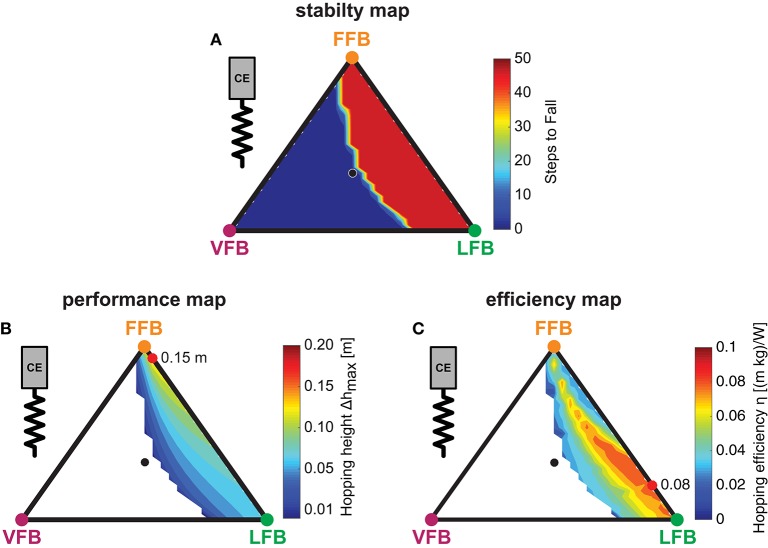
Sensor-motor maps: Influence of blended feedback pathways on hopping stability, performance and efficiency (*y*_0_ = 1.05 m, *G* = 1, *PreStim* = 0.01, ε_*moderate*_ = 0.03, rigid ground). Global maxima are visualized by red points. Every point within the triangle represents a unique combination of the three feedback pathways. The larger the distance of a point to a corner (e.g., LFB) the smaller the contribution of that feedback pathway in the blended signal (see Figure 3 for explanation of triangles). **(A)** Stability map: steps to fall (max = 50), **(B)** Performance map: maximum hopping height (Δ*h*_*max*_) and **(C)** efficiency map: hopping efficiency (η).

The blended feedback pathways produced both stable and unstable motions (Figure 5A). Motions of stable hopping (more than 50 hops) were found for compositions of FFB and LFB with small proportions of VFB. Here, a balanced composition of FFB and LFB resulted in greater stability (with respect to higher VFB proportion) compared to predominant FFB. A thin envelope of transitions (between 1 and 49 steps) was observed representing a distinct margin of stable and unstable areas.

#### 3.2.2. Hopping performance

The performance map (Figure 5B) shows the maximum hopping height (Δ*h*_*max*_) for all feedback compositions of steady-state motions where only stable predictions were considered. The contours show greater hopping heights for smaller proportions of VFB. In areas close to unstable solutions, the maximum hopping height (maximum vertical displacement of COM) was just above the leg length leading to smooth transitions from unstable (no hopping) to slight hopping patterns. Thus, the energy level of the system (*E*_*system*_) gradually increased when VFB was reduced. Compared to LFB, high proportions of FFB performed better, and higher hopping heights occurred closer to pure FFB. A composition of FFB and LFB (but not VFB) produced maximum performance (see red point in Figure 5B). Although, compared to individual contributions (e.g., pure FFB or LFB) hopping performance was amplified by blending multiple sensory pathways, the pathway-specific feature enabling motion performance (Geyer et al., 2003) was found for dominant FFB.

#### 3.2.3. Hopping efficiency

To identify feedback compositions resulting in hopping patterns that required less metabolic resources than others, the energetic relation of output and input: $\eta =\frac{\Delta h_{max}}{{\bar{M}}_{eff}}$ was used. In the topology of the efficiency map (Figure 5C), efficient motions were predicted in areas with dominant LFB and only small proportions of VFB (below 0.2), and a small band of efficient hopping patterns evolved. The spectrum of this band ranged from small proportions of FFB to pure LFB and gradually spread toward pure LFB. The most efficient feedback blending was found for a combination of small FFB, dominant LFB and no proportion of VFB (see maximum). Because VFB resulted in lower hopping heights (Figure 5B), VFB also reduced the hopping efficiency (η). Moreover, only moderate hopping heights led to most efficient hopping (Figure 5C). For the used metabolic model, hopping efficiency increased if the amount of positive work used for propulsion (and consequently hopping height) was reduced. Higher proportions of LFB led to lower force (and also work) production during late stance caused by a reduced activation signal in late stance due to the length offset (Figure 4B). In areas of higher hopping heights (dominant FFB and minor VFB), the metabolic model predicted a high metabolic effort leading to low efficiency values. As observed for hopping performance, the most efficient hopping pattern was found for a fusion of sensory pathways.

#### 3.2.4. Hopping motion

**Figure 6:**
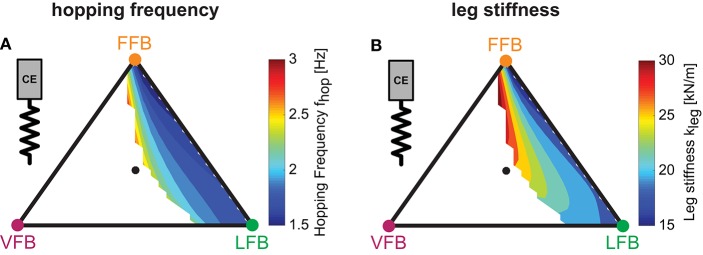
Predicted hopping motions: Influence of blended feedback pathways on hopping frequency and leg stiffness (*y*_0_ = 1.05 m, *G* = 1, *PreStim* = 0.01, ε_*moderate*_ = 0.03, rigid ground). Every point within the triangle represents a unique combination of the three feedback pathways. The larger the distance of a point to a corner (e.g., LFB) the smaller the contribution of that feedback pathway in the blended signal (see Figure 3 for explanation of triangles). **(A)** Hopping frequency (*f*_*hop*_) and **(B)** leg stiffness (*k*_*leg*_).

To evaluate and compare the predicted hopping motions with human hopping data (where possible), we calculated biomechanical parameters of the motions. Hopping frequencies ranged from 1.5 to 3.0 Hz (Figure 6A). Higher frequencies were found for higher VFB and hence in areas of smaller hopping heights (Figure 5B). The effective leg stiffness of our hopping model ranged from 15 to 30 kN/m (Figure 6B). This parameter depended mostly on the relation of VFB and LFB but was slightly influenced by increases in FFB (see vertical contour lines). Thus, the model produced motions of the best performing and most efficient compositions at small hopping frequencies (around 1.5 Hz) and low leg stiffness (around 15 kN/m).

### 3.3. Robustness of *Sensor-motor maps*

To explore the robustness of the *sensor-motor maps*, the effects of parameter variations of the model configuration (tendon elasticity ε_*ref*_, body mass *m*, segment lengths *l*_*S*_) and the environment (ground compliance *k*_*ground*_) were analyzed. Moreover, we investigated the parametric sensitivity of the model to variations of feedback and model parameters.

#### 3.3.1. Tendon elasticity changes

**Figure 7:**
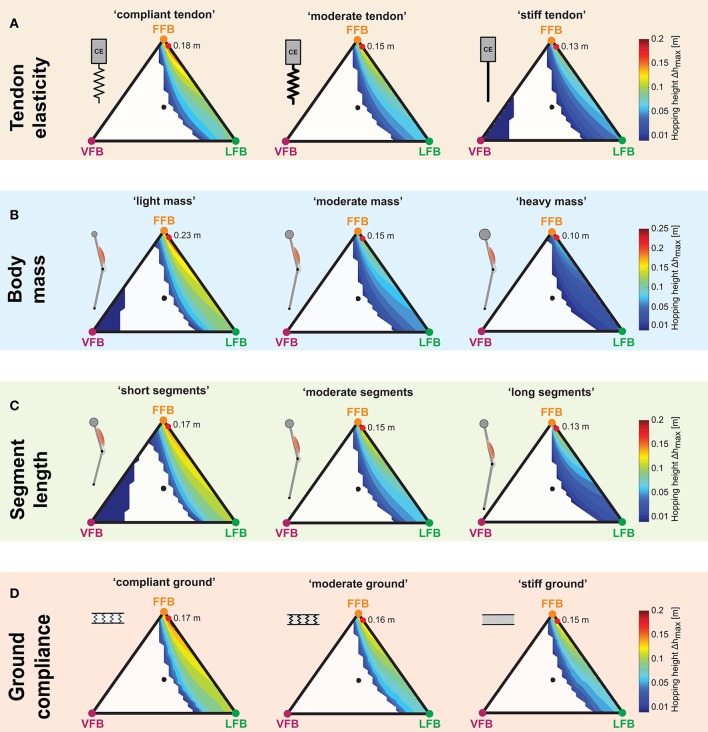
Influence of parameter variations on performance maps: Maximum hopping height (Δ*h*_*max*_) for blended feedback signals (*y*_0_ = 1.05 m,*G* = 1, *PreStim* = 0.01). Global maxima are visualized by red points. Every point within the triangle represents a unique combination of the three feedback pathways. The larger the distance of a point to a corner (e.g., LFB) the smaller the contribution of that feedback pathway in the blended signal (see Figure 3 for explanation of triangles). **(A)** Tendon elasticity changes: “compliant tendon” (ε_*compliant*_ = 0.05), “moderate tendon” (ε_*moderate*_ = 0.03), “stiff tendon” (ε_*stiff*_ = 0.01); **(B)** body mass changes: “light mass” (*m*_*light*_ = 64 kg), “moderate mass” (*m*_*moderate*_ = 80 kg), “heavy mass” (*m*_*heavy*_ = 96 kg); **(C)** segment length changes: “short segments” (*l*_*S,short*_ = 0.4 m), “moderate segments” (*l*_*S,moderate*_ = 0.5 m), “long segments” (*l*_*S,long*_ = 0.6 m); and **(D)** ground compliance changes: “compliant ground” (*k*_*compliant*_ = 100 kN/m), “moderate ground” (*k*_*moderate*_ = 500 kN/m), “stiff ground” (*k*_*stiff*_ = 9, 999 kN/m).

The three performance maps of altered elasticity of the serial elastic element showed only slight differences (Figure 7A). For all three tendon elasticity configurations, the size and location of stable hopping patterns were consistent, and smooth transitions from unstable to stable hopping patterns (with only small hopping heights) were predicted. While the topology of the performance maps remained similar (compared to the “moderate tendon”), the level of the predicted hopping height changed. The greatest hopping heights were found for a more compliant tendon and gradually decreased for stiffer configurations. Maximum hopping heights for each configuration ranged from 0.18 m (“compliant tendon”) to 0.15 m (“moderate tendon”) to 0.13 m (“stiff tendon”) and were found for consistent feedback compositions. For the stiffest elasticity (ε_*stiff*_ = 0.01), a second margin of stable solutions for high proportions of the VFB evolved. However, these feedback compositions resulted in hopping patterns just above the leg length (see also Figure 4C).

#### 3.3.2. Body mass changes

For all body mass configurations, performance map regions of stable hopping emerging for FFB and LFB remained similar (Figure 7B). A reduction of the body mass was predicted to result in higher maximum hopping height while the blending location of the most performant hopping patterns did not change. For the light mass, stable hopping patterns were found for dominant VFB.

#### 3.3.3. Segment length changes

The *sensor-motor map* topology remained similar for changes in the leg geometry (Figure 7C). Only for short segment lengths, VFB resulted in stable hopping patterns (with small hopping heights). Motions with the highest performance were found for a consistent sensory pathway blending, and the performance level increased with decreasing segment lengths.

#### 3.3.4. Ground compliance changes

Similar to the other parameter variations, changes in ground stiffness only minimally influenced regions of stable solutions (Figure 7D). Steady-state hopping heights decreased with increasing proportions of VFB (for all three ground configurations). Thus, maximum hopping heights were found for no proportions of VFB and dominant FFB, and decreased with decreasing ground compliance. The location of the maxima was consistent for different ground compliance and changes in the other parameter variations. The topology of the performance maps remained similar.

#### 3.3.5. Sensitivity of the model

We evaluated the sensitivity of specific model and feedback parameters to predicted hopping performance (Δ*h*_*max*_) and hopping efficiency (η) for seven feedback compositions (*S*_1_(*F*) to *S*_7_(*F,L,V*), Figure 3). Correlation coefficients (*r*), *p*-values and the standardized regression coefficients (β) are shown in Table 3. While pure VFB (*S*_3_(*V*)) did not generate any stable hopping pattern, for FFB (*S*_1_(*F*)) and LFB (*S*_2_(*L*)) 958 out of 1,000 simulations resulted in stable hopping. For both individual feedback pathways, hopping height was moderately influenced by the feedback signal time delay (Δ_*S*_): β = 0.637 (*r* = 0.585, *p* < 0.01, FFB) and β = 0.531 (*r* = 0.521, *p* < 0.01, LFB). Also, moderate correlations between hopping efficiency and reference strain (ε_*ref*_) were found for FFB (β = 0.628, *r* = 0.643, *p* < 0.01) and LFB (β = 0.431, *r* = 0.494, *p* < 0.01). For all feedback compositions, the model was most sensitive to the feedback signal time delay and the reference strain of the serial elastic element. Other feedback parameters such as gains (*G*_*i*_), offsets (*L*_*off*_, *V*_*off*_), the pre-stimulation bias (*PreStim*) or the model parameters segment length (*l*_*S*_) and the body mass (*m*) did not result in moderate or high correlations.

**Table 3:** Results of the sensitivity analysis.

**Correlations *r***	***S***_**1**_(***F***)		***S***_**2**_(***L***)		***S***_**4**_(***F**, **L***)		***S***_**5**_(***L**, **V***)		***S***_**6**_(***F**, **V***)		***S***_**7**_(***F**, **L**, **V***)	
***(regression***	**(*n* = 958)**		**(*n* = 958)**		**(*n* = 808)**		**(*n* = 733)**		**(*n* = 959)**		**(*n* = 950)**	
***coefficients β)***	**Δ*h*_*max*_**	**η**	**Δ*h*_*max*_**	**η**	**Δ*h*_*max*_**	**η**	**Δ*h*_*max*_**	**η**	**Δ*h*_*max*_**	**η**	**Δ*h*_*max*_**	**η**
*G*_*F*_	−0.083^**^	−0.442^**^	–	–	0.115^**^	0.43	–	–	0.037	−0.084^**^	0.100^**^	−0.045
	*(−0.111)*	*(-0.450)*	–	–	*(0.127)*	*(0.066)*	–	–	*(0.120)*	*(0.005)*	*(0.154)*	*(0.020)*
*G*_*L*_	–	–	0.016	−0.031	−0.022	−0.011	0.040	0.110^**^	–	–	0.010	0.068^*^
	–	–	*(0.004)*	*(−0.052)*	*(−0.021)*	*(−0.008)*	*(0.016)*	*(0.052)*	–	–	*(0.007)*	*(0.057)*
*L*_*off*_	–	–	0.198^**^	0.428^**^	0.350^**^	0.309^**^	0.097^**^	0.224^**^	–	–	0.138^**^	0.146^**^
	–	–	*(0.216)*	*(0.396)*	*(0.538)*	*(0.447)*	*(−0.157)*	*(0.104)*	–	–	*(0.116)*	*(0.117)*
*G*_*V*_	–	–	–	–	–	–	0.038	−0.304^**^	−0.079^*^	−0.246^**^	−0.060	−0.384^**^
	–	–	–	–	–	–	*(0.083)*	*(−0.314)*	*(−0.078)*	*(−0.234)*	*(−0.086)*	*(−0.414)*
*V*_*off*_	–	–	–	–	–	–	0.021	0.224^**^	0.007	−0.004	0.096^**^	0.050
	–	–	–	–	–	–	*(−0.088)*	*(−0.014)*	*(0.008)*	*(0.009)*	*(0.048)*	*(0.002)*
*PreStim*	−0.483^**^	−0.190^**^	0.198^**^	−0.014	−0.046	−0.009	−0.028	−0.051	−0.036	−0.040	−0.006	−0.003
	*(-0.530)*	*(-0.231)*	*(−0.028)*	*(−0.039)*	*(−0.049)*	*(−0.019)*	*(0.0001)*	*(−0.005)*	*(−0.007)*	*(−0.030)*	*(−0.020)*	*(−0.023)*
Δ_*S*_	0.585^**^	0.442^**^	0.521^**^	0.325^**^	0.426^**^	0.233^**^	0.677^**^	0.569^**^	0.837^**^	0.707^**^	0.737^**^	0.523^**^
	*(0.637)*	*(0.500)*	*(0.531)*	*(0.304)*	*(0.624)*	*(0.414)*	*(0.740)*	*(0.463)*	*(0.843)*	*(0.709)*	*(0.731)*	*(0.510)*
ε_*ref*_	0.287^**^	0.643^**^	0.119^**^	0.494^**^	0.379^**^	0.670^**^	0.268^**^	0.528^**^	0.287^**^	0.504^**^	0.246^**^	0.510^**^
	*(0.338)*	*(0.628)*	*(0.084)*	*(0.431)*	*(0.429)*	*(0.701)*	*(0.238)*	*(0.492)*	*(0.306)*	*(0.480)*	*(0.264)*	*(0.492)*
*l*_*S*_	0.240	−0.010	0.023	−0.028	−0.22	−0.012	0.017	0.031	0.007	0.032	0.008	−0.003
	*(0.029)*	*(−0.024)*	*(0.026)*	*(−0.005)*	*(−0.008)*	*(−0.008)*	*(0.018)*	*(0.004)*	*(−0.018)*	*(−0.006)*	*(0.001)*	*(0.025)*
*m*	−0.304^**^	−0.014	−0.246^**^	−0.123^**^	−0.313^**^	−0.196^**^	−0.225^**^	−0.092^*^	−0.233^**^	−0.140^**^	−0.303^**^	−0.105^**^
	*(−0.411)*	*(−0.107)*	*(−0.263)*	*(−0.112)*	*(−0.360)*	*(−0.231)*	*(−0.298)*	*(−0.077)*	*(−0.272)*	*(−0.151)*	*(−0.313)*	*(−0.112)*
^*^p < 0.05; ^**^p < 0.01												

*For each specific composition, n depicts the number of stable simulations (max(n) = 1, 000). Correlations r (Spearman-Rho) and standardized regression coefficients β (in parentheses) of predictors on the maximum hopping height (Δh_max_) and the hopping efficiency (η). Composition S_3_(V) (pure VFB) did not result in any stable hopping simulations (n = 0). High (r≥0.7 or r ≤ −0.7) and very significant (p < 0.01) correlations are highlighted. (y_0_ = 1.05 m, G = 1, PreStim = 0.01, ε_stiff_ = 0.03, rigid ground)*.

* p < 0.05;

** *p < 0.01. The italic font corresponds to the standartized regression coefficients, the bold font highlights high correlation and very significant results*.

#### 3.3.6. Muscle-tendon interaction

**Figure 8:**
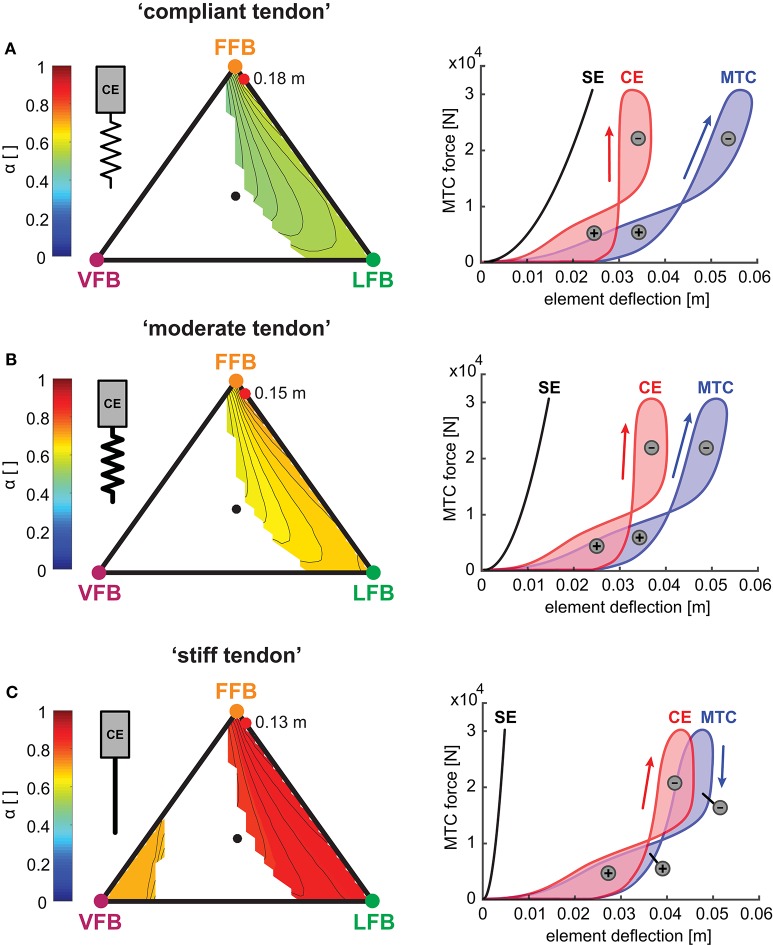
Left side: Influence of serial elasticity on muscle interaction maps: Maximum work ratio of CE to whole MTC (α) for blended feedback pathways (*y*_0_ = 1.05 m,*G* = 1, *PreStim* = 0.01, rigid ground). Global maxima of hopping heights are visualized by red points. Every point within the triangle represents a unique combination of the three feedback pathways. The larger the distance of a point to a corner (e.g., LFB) the smaller the contribution of that feedback pathway in the blended signal (see Figure 3 for explanation of triangles). **(A)** “Compliant tendon” (ε_*compliant*_ = 0.05), **(B)** “moderate tendon” (ε_*moderate*_ = 0.03) and **(C)** “stiff tendon” (ε_*stiff*_ = 0.01). Right side: Influence of serial elasticity on individual work loops of CE (red), SE (black) and MTC (blue) for maximum hopping heights. Positive (energy generation) and negative (energy dissipation) work loops are indicated by positive and negative signs.

To further explore the robustness of the *sensor-motor maps* we investigated the muscle-tendon interaction because the elasticity of the SE also influenced the interplay of the CE and the SE. The muscle interaction maps in Figure 8 show the calculated index α for each tendon configuration describing the relation of maximum work generated by the CE to the whole MTC. While values of α were mostly determined by the relation of VFB and LFB (see vertical contours), the map topologies were only slightly influenced by changes in serial elasticity. α values decreased with increasing tendon compliance, and α values at the location of maximum hopping heights ranged from 0.9 (“stiff tendon”) to 0.75 (“moderate tendon”) to 0.57 (“compliant tendon”). The related work loops show the detailed interplay of CE and SE for simulations that predicted the highest hopping heights (red points). At touch-down, the MTC was stretched by a low force. The MTC generated forces (feedback response) while being stretched (eccentric contraction) which led to negative work loops. During leg extension, the MTC shortened during force generation (concentric contraction) and produced a positive work loop. More compliant tendon resulted in slightly higher MTC deflections and less lengthening of the CE. Because maximum MTC forces did not change with different elasticity, a stiffer configuration caused less deflection and reduced the energy recoil. The energy stored in the SE decreased from 728 J (“compliant tendon”) to 437 J (“moderate tendon”). For a maximal hopping height of 0.13 m, the “stiff tendon” stored the least amount of energy with 142 J.

## 4. Discussion

This simulation study investigated the composition of afferent feedback pathways for generating a repulsive leg response in hopping. Therefore, a neuromuscular reflex model (Geyer et al., 2003) was extended by blending length (LFB), force (FFB) and velocity feedback pathways (VFB) of one anti-gravitational leg extensor muscle. *Sensor-motor maps* were derived to evaluate the predicted motion with respect to stability, performance and efficiency. The topology of the *sensor-motor maps* was further evaluated for different tendon elasticity, body mass, segment lengths and ground compliances. Below, we first highlight the key insights gained by our hopping model, which will then be discussed in more detail.

1. Different feedback pathways had specific functional contributions: Both FFB and LFB pathways enabled hopping. FFB resulted in largest hopping heights, LFB enhanced hopping efficiency (ratio of hopping height to metabolic effort), and VFB had the ability to disable hopping (also in combination with FFB and LFB). These pathway-specific responses established *sensor-motor maps* with function-selecting and -tuning pathways in hopping (Figure 5).
2. For the tested case, the topology of these *sensor-motor maps* as well as the location of functionally optimal compositions was invariant to altered system designs (tendon elasticity, body mass, segment lengths, Figures 7A–C) or environmental changes (ground compliance, Figure 7D). Thus, in our model the neuromuscular feedback system relied on a consistent topology of feedback compositions.

The modeling framework presented here can be used to establish relations to biomechanical (loco-)motion concepts (e.g., preflex Loeb, 1995; Brown and Loeb, 2000) and template models (Full and Koditschek, 1999) and to explore the capacity and physiological limitations of the biological neuromuscular system (Pearson et al., 2006), e.g., due to signal delays or muscle dynamics. Moreover, neuromuscular simulation models can be validated, improved and used for different applications, for instance to derive model-based experimental designs for investigating human or animal motor control. The results of our study should be confirmed by experimental studies in biological systems.

### 4.1. Different feedback pathways have different functional contributions

We found pathway-specific features that resulted in different characteristics of the hopping motion. Firstly, FFB was the dominant feedback pathway to produce high hopping heights and thus high hopping performance. Previous studies reported that the combination of the muscle force-velocity-relationship and positive force feedback (FFB) produced stable and high-performing motions (Prochazka et al., 1997b,a; Geyer et al., 2003; Haeufle et al., 2012). In contrast to a combination of the force-length-relationship (*f*_*l*_) and negative LFB (which would result in similar behavior compared to FFB Prochazka and Yakovenko, 2002), negative LFB did not provide self-stabilizing behavior (Haeufle et al., 2010). An elastic structure or the *f*_*l*_ helps to achieve energy efficient and spring-like behavior, but does not generate energetically stable hopping on its own (Haeufle et al., 2010). However, experimental evidence for functional force feedback of ankle extensor activity to generate a repulsive leg function has been found for walking cats (Donelan and Pearson, 2004a,b) and humans (Grey et al., 2007; af Klint et al., 2010), but may be different for hopping motions.

Secondly, efficient hopping was found for areas of moderate hopping height in which LFB is dominant. This occurred because the feedback suppression of the length offset (e.g., through fusimotor drive) reduced the positive work production of the CE in late stance phase also reported by Geyer et al. (2003) resulting in high metabolic effort. In our simulations, this suppression could be used to fine-tune the hopping efficiency by determining the length-dependent activation of the CE during stretch-shortening cycle (SSC) (Gollhofer, 2003). This influenced the force-lengthening characteristic of the MTC and thus the energy recoil of the serial elastic element (with the used tendon elasticity). Raburn et al. suggested that proprioceptive information is used to adjust a periodic bouncing motion pattern to achieve energetically optimal patterns (Raburn et al., 2011). If afferent pathways of muscle spindles are blocked by an ischemia blockage, the patterns are less adapted (Raburn et al., 2011). It was argued, that the observed effect was most likely caused by an update of the internal model for planning the motion. Nonetheless, implications of direct activation pattern changes as considered in our model could not be ruled out (Dean, 2012). It seems reasonable that reflex contribution directly influences the SSC (e.g., through generating “muscular stiffness” Nichols and Houk, 1976; Gollhofer, 2003) and thus the hopping efficiency (Komi, 2003).

Surprisingly, blending dominant VFB and other feedback pathways did not result in a higher performance, but lead to substantial losses in hopping height resulting from an early rise of the activation signal. Next to this extensive force generation (during leg compression) the CE remained less stretched during stance (force-length-relationship below 0.2) leading to lower push-off forces and hopping performance. This shift of the CE operation point was also found in the simulation study by Robertson and Sawicki (2014) where higher muscle stimulation frequencies (earlier stimulation onset) and magnitudes resulted in less CE lengthening. In our model, this effect was stronger than the resultant performance increase associated with an increased proportion of FFB. Thus, we found a function disabling behavior for compositions with dominant VFB and small feedback signal time delays. These results indicate that VFB might function as the primary regulator of the system energy in hopping complementing the previous suggestion that FFB might be used to control the energy state of the system (Geyer et al., 2003). McDonagh and Duncan (McDonagh and Duncan, 2002) provided evidence for the contribution of velocity-sensitive afferent signals in their experimental study of landing motions. Increased electromyographic (EMG) amplitudes of gastrocnemius and rectus femoris muscles were found for increased ankle and knee joint velocities at touchdown (due to different landing heights) during false floor landings compared to expected grounds (McDonagh and Duncan, 2002). For hopping, it might be necessary to delay or inhibit the VFB for example by presynaptic inhibition to functionally enable FFB or LFB pathways. Such inhibition of VFB in hopping were indicated by results by Voigt et al. (1998) who reported a negative correlation between peak stretch velocity and EMG amplitude of the soleus muscle. However, the disabling function of the VFB may not be transferable to other motion tasks such as walking. Because such feedback mechanisms are strongly task and phase dependent the contribution of a feedback pathway may have opposite effects for different motion tasks, e.g., in walking and standing (Pearson and Collins, 1993; Donelan and Pearson, 2004b). In a biological system this mechanism might be superimposed or modulated by fusimotor action (Prochazka and Ellaway, 2012) or descending signals (see section 4.3). Thus, further research is warranted to identify the task-specific role of VFB in generating appropriate leg extensor muscle activation in humans and animals.

### 4.2. Robustness of *Sensor-motor maps*

Regions of stable and unstable hopping motions were only minimally influenced by the changes in tendon stiffness, body mass, segment lengths and ground compliance. Stable solutions for pure VFB were found for “light mass,” “short segments” or “stiff tendon” and resulted in very low hopping performance. This result is in agreement with previous studies where VFB produced stable hopping only in the absence of serial compliance (Geyer et al., 2003; Haeufle et al., 2012). More compliance resulted—as expected—in greater hopping heights and more efficient hopping patterns. Similar results were found in experimental and computational studies (Anderson and Pandy, 1993; Kubo et al., 1999; Bobbert, 2001; Nagano et al., 2004). While the level of hopping height was influenced by all altered configurations, the topology of these maps was not affected. Interestingly, we found that for all parameter variations a consistent pathway composition resulted in the maximum hopping performance suggesting a unified *sensor-motor map* topology with consistent optimal solutions. Apart from this isolated observation, the (multidimensional) sensitivity analysis also revealed only small to moderate dependencies of the predicted hopping height and hopping efficiency for changes in tendon elasticity, body mass or segment lengths.

Our results indicate that *sensor-motor maps* are robust against these morphological changes. Previous studies showed that the elastic leg function is highly determined by the interplay of the compliant tendomuscular system and the neuronal control system (Nichols and Houk, 1976; Lin and Crago, 2002; Gollhofer, 2003; Taube et al., 2012; Robertson and Sawicki, 2014). In our simulations, a more compliant tendon was able to store and release more energy during hopping agreeing with other studies (Anderson and Pandy, 1993; Bobbert, 2001). Moreover, the model predicted decreasing α values with increasing tendon compliance (increased tendon lengthening) revealing a compensatory behavior of the CE by stiffening (producing equal forces with less deflection). Such behavior was observed by experimental studies, where changes in ground stiffness lead to adaptations of the leg stiffness, such that the total stiffness, consisting of leg and ground, remains similar (Ferris and Farley, 1997; Moritz and Farley, 2004; van der Krogt et al., 2009). A similar stiffness adaptation was found for hopping with a passive ankle joint orthesis acting in parallel to the ankle (Ferris et al., 2006). For our model, this effect may be accomplished by the stabilizing function of the non-linear muscle properties (van Soest and Bobbert, 1993; Moritz and Farley, 2004; van der Krogt et al., 2009; Haeufle et al., 2010) as the feedback pathway composition remained the same for all three conditions. These “exploit mechanics” or “preflex” function (Loeb et al., 1999) may serve as a selector (functional filter) to offer favorable solutions for the neural motor control system (*sensor-motor maps*) that allow to learn its simple (Loeb, 1995) and consistent topology.

### 4.3. Integration of spinal reflexes and feed-forward control

Although we did not consider supra-spinal motor commands, we would like to reflect our results on their functional integration with feed-forward commands. Our model suggests that the neuromuscular feedback system alone could generate appropriate adjustments of the muscle activation to permit a fine-tuning of hopping motions. In addition to our results, previous experimental studies revealed the importance of pre-planned feed-forward commands in hopping and drop jumps. Descending commands may contribute during the early contact phase (Zuur et al., 2010) and toward push-off (Taube et al., 2008). Accordingly, the EMG activity of ankle extensor muscles (such as the soleus) was found to be pre-programmed and adjusted dependent on the task (e.g., jumping or landing) (Leukel et al., 2008a, 2012) and with respect to the timing of the touch-down (Santello and McDonagh, 1998; McDonagh and Duncan, 2002). Integrating feed-forward commands would certainly influence the results presented in this study. For example, pre-planned motor commands could compensate the repressive behavior following dominant VFB. Such descending commands could be superposed to our blended feedback signal (as *PreStim* in our model) (Taube et al., 2012) or adapt and suppress the afferent gating (*G* in our model), for instance by presynaptic inhibition (McDonagh and Duncan, 2002; Leukel et al., 2008b,a).

The motor control system is likely to rely more on afferent feedback in the case of misplanned motions (if perturbations occur) or if knowledge about the environment is uncertain and planning is difficult (Donelan and Pearson, 2004b). The contribution of afferent feedback pathways may increase under these conditions (McDonagh and Duncan, 2002). A function specific fine-tuning of hopping motions with respect to different movement targets or cost functions (e.g., performance, efficiency), as found for our model, would support the generation of appropriate activation patterns in such conditions. Therefore, supra-spinal centers might plan an appropriate blending of the afferents (setting of λ_*F,L,V*_) before touch-down. For example, in our model shifting from targeting hopping performance to hopping efficiency could be moderated by fading from dominant FFB to dominant LFB (see Video in Supplementary Material). By comparing predicted and actual afferents (Wolpert et al., 1995; McDonagh and Duncan, 2002), the overall feedback gain (in our model *G*) might increase if deviations and errors are detected. If so, the pre-setting of the afferent blending permits a fast and function-oriented contribution (performance, efficiency) of feedback responses. Another advantage would be the reduced control effort due to the low-dimensionality of the blending (setting of λ_*F,L,V*_). However, higher centers must be able to learn such feedback blending even in the case of sensorial and mechanical perturbations. Thus, in order to be functionally useful, the solution space of possible feedback compositions (as shown in the *sensor-motor maps*) must follow a simple and consistent topology (Loeb, 1995). Indeed, we found compact and robust topologies that were invariant to changes of morphological design or environmental parameters with respect to motion stability and optimal compositions. Nonetheless, we can only derive hypotheses about a potential integration of our feedback model and feed-forward commands. The discussion presented here warrants validation and support from experimental studies.

### 4.4. Comparison of the model to human hopping

The neuromechanical hopping model (with a reference strain of ε_*moderate*_ = 0.03, “moderate” mass, “moderate” segment lengths and rigid ground) produced similar hopping motions as those of human hopping. A high effective stiffness of the leg was found for high hopping frequencies. While similar results have been reported in experimental studies (Farley and Morgenroth, 1999; Riese et al., 2013), other studies reported higher leg stiffness values ranging from 30 to 55 kN/m (Farley et al., 1991; Hobara et al., 2011; Kuitunen et al., 2011). However, hopping frequency and leg stiffness were in reasonable ranges and changed accordingly (Rapoport et al., 2003). Moreover, our model predicted increasing hopping performance with increasing tendon elasticity agreeing with results of experimental studies (Kubo, 2005; Fukashiro et al., 2006) and other simulation studies (Anderson and Pandy, 1993; Bobbert, 2001; Nagano et al., 2004). Although this study utilises a highly simplified model structure the model predicted the basic dynamics (e.g., hopping frequency and leg stiffness) of human hopping. We thus consider this model a valid simplification for the scope of this study.

### 4.5. Model limitations

We chose a rather simplistic model to integrate multiple sensory pathways at the elementary sensor-motor-level to determine how individual reflex pathways of muscle force (FFB), fiber length (LFB) and velocity (VFB) can support—in isolation and in combination—the repulsive leg function during the stance phase of hopping. By blending individual sensory pathways, we investigated the capacity of the neuromuscular feedback system to generate goal-directed motions. These simplifications may have influenced our results.

We selected a bouncing task (one dimensional hopping) as primary locomotor function. The functional contribution of the different feedback pathways predicted for hopping will most likely be different for other motion tasks. The hopping model of Geyer et al. (2003) utilized one anti-gravitational leg extensor muscle representing all involved muscles during the stance phase of hopping. The foot segment and ankle joint were neglected possibly influencing the overall leg stiffness of the model because the ankle joint was found to be the main contributor for leg stiffness modulations at higher hopping frequencies (above 2.2 Hz; Farley et al., 1998; Farley and Morgenroth, 1999; Hobara et al., 2011). Nonetheless, the model of Geyer et al. (2003) and our simulations generated biomechanically reasonable results. In addition, we simplified the distributed mass of the human body to a point mass neglecting the effects of wobbling masses and their influence on impact dynamics (Seyfarth et al., 1999; Schmitt and Günther, 2011). This helped to focus on the muscle-tendon interaction and the functional contribution of different feedback pathways. The model of the MTC did not consist of a parallel elastic element that could (additionally to the SE) store and release energy in SSC (Anderson and Pandy, 1993; Lindstedt et al., 2002; Robertson and Sawicki, 2014) when the CE is stretched beyond its optimal length (van Soest and Bobbert, 1993). However, in our simulations, the CE did not operate above its optimal length. Hence, we argue that it is tenable to neglect the parallel elastic element in this particular case. In addition, jumping simulation models by Anderson and Pandy (1993) and Seyfarth et al. (2000) showed only insignificant contribution of the parallel elastic elements. Moreover, while in our study damping within the MTC was neglected, a damping element has also been found to be negligible for hopping (Rapoport et al., 2003).

The used neuromuscular feedback model is a highly simplified representation of the complex biological network. Sensory signals were handled as ideal, averaged and analogue physical quantities without frequency modulation and sensory or signal noise. By using simple delays, offsets and gains, we only considered a highly simplified neural processing of the monosynaptic feedback pathways. In particular, no time-variant feedback gains (Pearson, 1995; Pearson et al., 1998) or other sensory signals such as joint position and velocity, mechanoreceptors or cutaneous receptors were considered. Because we found moderate correlations for the feedback time delay, feedback-specific delays (Prochazka et al., 1997b) will certainly influence the performance and efficiency of the model. Possible causes of afferent gain changes as discussed in Sreenivasa et al. (2015) were not investigated here. All these factors were simplified and neglected for the sake of simplicity and comprehensibility (Full and Koditschek, 1999; Brown and Loeb, 2000; Pearson et al., 2006). For this study, it was important to separate and isolate the pathway and task-specific effects in the frame of the mechanical structure and muscle mechanics. Previous studies of similar model complexity (Kuo, 2002; Geyer et al., 2003; Haeufle et al., 2010, 2012) not only demonstrated realistic motions but also elucidated the functional roles of the different contributors of feedback, feed-forward or muscle properties.

### 4.6. Outlook and future directions

Based on the results of this study, the robustness of the system against mechanical or sensory perturbations can be investigated. From a control point of view, combinations of multiple sensory pathways or information channels about the system state will result in more robust and more precise estimations of the system state (Donelan and Pearson, 2004b; Green and Angelaki, 2010). Comparing effect sizes resulting from muscle properties (Haeufle et al., 2010), feedback blending and an integration of feed-forward controls (Kuo, 2002; Haeufle et al., 2012) is of high interest. In a next step, we will expand our models to other motions tasks (e.g., running and walking) and underlying locomotor subfunctions (Sharbafi and Seyfarth, 2017) such as swing and balancing (Seyfarth et al., 2012; Sharbafi et al., 2017). For these scenarios, other feedback pathways, e.g., from vestibular organs or as suggested by Song and Geyer (2015), may be used to examine the generalisability of the *sensor-motor maps*.

Moreover, we would like to explore the use of this type of neuromuscular model as non-invasive distinguishing tool for two purposes: (1) to further explore the mechanisms and interactions of mechanical, neuromuscular and sensory templates and (2) for a model-based design of experimental protocols and settings (e.g., perturbation profiles). While computational modeling approaches help to investigate underlying principles of locomotion, they could potentially help to predict the value and usefulness of experimental settings. Such a model-based identification of experimental settings might improve future experimental designs.

## 5. Conclusion

The novel *sensor-motor maps* provide a tool for analysing human (and animal) motor control strategies and investigating how the biological neural control system recruits function-specific sensor-motor pathways. The maps of muscle force, fibre length and velocity pathways are predicted to be robust with respect to changes in body and environment mechanics (e.g., compliance).

In addition to central (or spinal) pattern generators, muscle-reflex based control circuits are able to generate adjustable cyclic motions by exploiting the musculoskeletal dynamics and gravity. We call these neuromechanical pattern generators (nmPG's). Accordingly, the sensory feedback pathways (e.g., positive force feedback) operate as an antagonist system to the mechanics of the body (muscles, segments) and gravity. The mechanical system is consequently not only the target of (neuronal) control but at the same time an essential part of pattern generating networks.

## Author contributions

CS and AS contributed to the design, execution and drafting of this work, and approved the final manuscript. CS implemented the computational model and conducted the simulation studies.

### Conflict of interest statement

The authors declare that the research was conducted in the absence of any commercial or financial relationships that could be construed as a potential conflict of interest.
